# ‘Hummingbird’ sign in progressive supranuclear palsy

**DOI:** 10.4103/0972-2327.53087

**Published:** 2009

**Authors:** Rakesh Shukla, Manish Sinha, Rajesh Kumar, Dilip Singh

**Affiliations:** Department of Neurology, CSM Medical University (erstwhile King George's Medical University), Lucknow - 226 003, India

Presence of the ‘hummingbird’ sign in brain MRI is an interesting radiological sign in the patients with progressive supranuclear palsy (PSP). In this article we report a patient of PSP who demonstrated the ‘hummingbird’ sign.

A 66-year-old male presented with slowness of activities and falls while walking; his symptoms had an insidious onset and had been gradually progressive over the last 3 years. He gave no history of tremulousness, forgetfulness, hallucinations, postural dizziness, or urinary incontinence. There was no history of a similar illness in his family.

**Figure 1:**
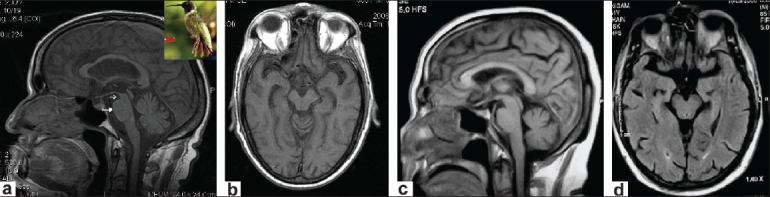
(a) Midsagittal T1W MRI brain showing atrophy of the midbrain tegmentum (open arrow) with a relatively preserved pons (closed arrow), resembling the head and body, respectively, of a hummingbird (inset). (b) Midbrain tegmentum atrophy is evident in axial T1W MRI of the brain. (c and d) Midsagittal and axial T1W MRI of the brain of a normal age and sex mathced control

On examination, supranuclear horizontal and vertical gaze palsies, axial rigidity, bradykinesia, and generalized hyperreflexia were present. The patient was diagnosed as probable PSP. Midsagittal T1-weighted MRI of the brain revealed atrophy of the midbrain tegmentum, with a relatively preserved pons; this gave an appearance resembling the head and body, respectively, of a hummingbird [Figure 1]. This is known as the ‘hummingbird’ sign.[1,2] Demonstration of the hummingbird sign on MRI is thought to be useful for establishing the diagnosis of PSP; it is reported to have a sensitivity of nearly 100%.[1] Patients with Parkinson's disease, multisystem atrophy, and corticobasal degeneration have no midbrain atrophy and therefore do not show this sign.
